# What is the role of lateral lymph node dissection in rectal cancer patients with clinically suspected lateral lymph node metastasis after preoperative chemoradiotherapy? A meta‐analysis and systematic review

**DOI:** 10.1002/cam4.2643

**Published:** 2020-04-30

**Authors:** Xuyang Yang, Shuo Yang, Tao Hu, Chaoyang Gu, Mingtian Wei, Xiangbing Deng, Ziqiang Wang, Zongguang Zhou

**Affiliations:** ^1^ Department of Gastrointestinal Surgery West China Hospital Sichuan University Chengdu China

**Keywords:** lateral lymph node, lateral lymph node dissection, meta‐analysis, neoadjuvant chemoradiotherapy, rectal cancer

## Abstract

**Background:**

Local lateral recurrence (LLR) in rectal cancer is increasingly becoming a significant clinical issue. Preoperative neoadjuvant chemoradiotherapy (nCRT) and lateral lymph node dissection (LLND)—when each approach is separately executed—cannot cure lateral lymph node metastasis (LLNM). Here, we performed a meta‐analysis to evaluate the efficacy of nCRT plus total mesorectal excision (TME) vs TME plus LLND after nCRT for rectal cancer.

**Methods:**

Standard databases (PubMed, Embase, MEDLINE, Cochrane Library, and Web of Science) were searched to identify all relevant studies comparing nCRT+TME and nCRT+TME+LLND. Data in the included studies were extracted, and intraoperative outcomes, postoperative complications, and oncological outcomes were evaluated.

**Results:**

Eight studies representing 1,896 patients (1,461 nCRT+TME vs 435 nCRT+TME+LLND) were included. We found that for patients with clinically suspected LLNM, the incidence of pathological LLNM was 27.8%, even after nCRT. LLND after nCRT was significantly associated with lower LLR (*P* = .02). Additional LLND yielded a longer operative time (*P* < .01) and increased the risk of urinary dysfunction (*P* < .01). Concerning other outcomes, no significant differences were identified between the two groups.

**Conclusion:**

This is the first meta‐analysis and systematic review of studies comparing nCRT+TME and nCRT+TME+LLND for rectal cancer patients. Although increasing operative time and the risk of urinary dysfunction (which might be ameliorated by minimally invasive procedures), the pooled results support the use of LLND after nCRT and TME for reducing LLR in patients with clinically suspected LLNM and provide another treatment option for high‐risk patients.

## Graphical abstract

Our meta‐analysis firstly confirmed that additional lateral lymph node dissection could further decrease local lateral recurrence in patients with clinically suspected lateral lymph node metastasis after neoadjuvant chemoradiotherapy followed by total mesorectal excision.

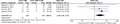

## 1 INTRODUCTION

Considerable controversy still exists in regard to the treatment of lateral lymph nodes (LLNs) in rectal cancer patients. It is well‐known that preoperative neoadjuvant chemoradiotherapy (nCRT) combined with total mesorectal excision (TME) is the standard treatment for locally advanced rectal cancer in Western countries, whereas prophylactic lateral lymph node dissection (LLND) has not been adopted for postoperative complications and does not offer any additional oncological benefits.^1^, ^2^ On the contrary, in Asian countries—especially in Japan—the latest guidelines still recommend that routine LLND is performed in patients with lower locally advanced rectal cancer for decreasing the risk of local recurrence (LR) and improving overall survival (OS).^3^

In recent years, increasing evidence has shown that local lateral recurrence (LLR) is a significant clinical issue, especially in patients with clinically suspected lateral lymph node metastasis (LLNM), due to a high risk of treatment failure with TME plus either nCRT or LLND.^4^, ^5^, ^6^ The most important recent changes in the management of LLN suggest that approaches between Western and Eastern countries are finally converging.^4^ LLND and nCRT for rectal cancer can be mutually beneficial.^7^ Surgeons in Japan have reconsidered the role of prophylactic LLND in rectal cancer, which resulted in the rate of LLND decreasing from 48.1% between 2010 and 2011 to 30.1% in 2012^8^; instead, they have started to adopt nCRT with indicated (or selective) LLND in patients with clinically suspected LLNM.^9^, ^10^ In Western countries, surgeons have also recently begun performing LLND, which is striking since this procedure was almost entirely neglected previously.^11^, ^12^

It is currently still unclear whether patients receive more benefits from additional LLND after TME following nCRT. Given the urgency of solving this issue and the limited number of cases in the literature, we performed this first meta‐analysis and systemic review to determine if patients with rectal cancer who adopted preoperative nCRT benefit from additional LLND.

## 2 METHODS

To ensure data quality, this meta‐analysis and systemic review were performed in line with recommendations from the Cochrane Collaboration.^13^ The Preferred Reporting Items for Systematic Reviews and Meta‐analysis (PRISMA) statement was also followed for this review.^14^

### 2.1 Search strategy and data sources

A systemic literature search was performed through PubMed, Ovid Embase, Ovid Medline, Cochrane Library, Web of Science, and ClinicalTrials to identify any potentially relevant studies comparing rectal cancer patients who underwent nCRT+TME vs patients who underwent nCRT+TME+LLND. There were no limits on languages, regions, or publication types. We also physical searched key journals and checked reference lists to identify additional appropriate studies.

The prespecified search terms (Medical Subject Headings terms and keyword terms) were grouped into the following four classifications: (a) “rectal cancer” terms (rectum/rectal; carcinoma/cancer/tumor/tumour/neoplasms); (b) “preoperative treatment” terms (neoadjuvant chemoradiotherapy/preoperative; radiotherapy/preoperative; chemoradiotherapy/neoadjuvant; preoperative chemoradiotherapy/neoadjuvant; treatment/neoadjuvant therapy); (c) “rectal resection” terms (total mesorectal excision/rectal; resection/extended resection/radical resection/low anterior resection/anterior resection); and (c)”lateral lymph node dissection” terms (lateral lymph node dissection/lateral pelvic lymph node dissection/lateral pelvic wall lymph node dissection/pelvic sidewall lymph nodes dissection/extended lymphadenectomy/pelvic lymphadenectomy/extended lateral pelvic lymph node dissection). The publication period for all included articles was 1946‐2019.

### 2.2 Eligibility criteria for including studies

Eligible studies for this meta‐analysis were those evaluating nCRT+TME vs nCRT+TME+LLND for rectal adenocarcinoma, including randomized controlled trials (RCTs) or prospective/retrospective cohort studies.

Studies were excluded if they met any of the following exclusion criteria: (a) specific types of literature including letters, reviews, commentaries, conference abstracts, or case reports; (b) involvement of other malignant tumors, such as urologic or gynecologic tumors; (c) cadaveric or animal studies; (d) difficulty in extracting data from published results; (e) single‐arm study design (eg, nCRT+TME only, TME+LLND only, or nCRT+TME+LLND only); (f) other surgical procedures included (eg, LLN sampling, hemipelvectomy, total pelvic resection, or sacrectomy); or (g) unavailable article.

### 2.3 Outcomes of interest

The primary outcomes of interest in this meta‐analysis were oncological benefits of additional LLND after nCRT and TME. The oncological considerations included the incidence of LR, LLR, pathological LLNM, OS, and disease‐free survival (DFS). The secondary outcomes of interest were in the form of safety considerations, including postoperative complications (anastomotic leakage, perineal/abdominal wound infection, bowel obstruction, and genitourinary dysfunction) and other intraoperative outcomes (blood loss and operative time). LR was defined as any tumor recurrence within the pelvic cavity, and distant recurrence was defined as any tumor metastasis outside of the pelvic cavity. Lateral recurrence was defined as any recurrence occurring in the lateral compartment. Postoperative complication was defined as any complication > Grade 1 according to the Clavien–Dindo classification.

### 2.4 Study selection

The four investigators (XYY, SY, TH, and CYG) independently searched and evaluated the titles, abstracts, and full‐text articles of all identified studies. In the event of any discrepancy, a consensual decision was made.

### 2.5 Data collection

A predefined extraction form was used to extract data from all eligible studies independently by two reviewers (XYY and SY). The information extracted included the authors, country, type of study, publication year, number of patients in each arm, population characteristics, tumor characteristics, indication for nCRT+TME or nCRT+TME+LLND, surgical procedures, and follow‐up times. We contacted the primary author if needed to acquire information not available in the manuscript.

### 2.6 Quality assessment

The modified Newcastle‐Ottawa Scale (NOS) was used to evaluate the methodological quality of the enrolled cohort studies.^15^, ^16^ The NOS score ranges from 0 to 9, and studies with a score ≥5 were assumed to be of high quality. The Jadad scoring system was used to assess the methodological quality of randomized controlled trials (RCTs).^17^ When the total for the Jadad score was ≥ 3, the RCT study was considered to be of high quality. Moreover, the methodological quality of each RCT was also assessed by the Cochrane Collaboration's risk‐of‐bias assessment tool.^13^

### 2.7 Statistical analysis

According to the recommendations from the PRISMA statement and the Cochrane Handbook for systematic reviews, Review Manager software (Version 5.3; Copenhagen: The Nordic Cochrane Centre, The Cochrane Collaboration, 2008) was used for statistical analysis by three reviewers (XYY, TH, and CYG). As we described previously, a random‐effect model was used to pool individual datasets for heterogeneity across studies. Dichotomous variables were analyzed by the Mantel‐Haenszel statistical method combined with the odds ratio (OR). Continuous variables were analyzed by weighted mean differences (MD) and are expressed as the mean ± standard deviation (SD). All variables were reported with 95% confidence intervals (CIs). If only the median, range, and sample size of the study were reported, the mean and SD were estimated based on the method described by Hozo et al^18^ Survival data were extracted from a Kaplan‐Meier curve, and hazard ratios (HRs) were used for the corresponding quantitative analysis.^19^ Heterogeneity across studies was assessed by the Cochran Q statistic (χ2 test) with I square test (I^2^). An I^2^ value >50% was considered to represent significant heterogeneity, and subgroup analysis was performed to explore the sources of heterogeneity, if possible. A *P*‐value ≤.05 was considered to indicate statistical significance. Additionally, sensitivity analysis was also conducted for the following subgroups: (a) studies including more than 20 patients in each group; (b) studies published in or after 2010; and (c) high‐quality studies. A narrative review was carried out due to less than three studies for each analysis or highly significant heterogeneity existing after subgroup analysis. A funnel plot was constructed to investigate possible publication bias.

## 3 RESULTS

### 3.1 Literature search and study selection

**Figure 1:**
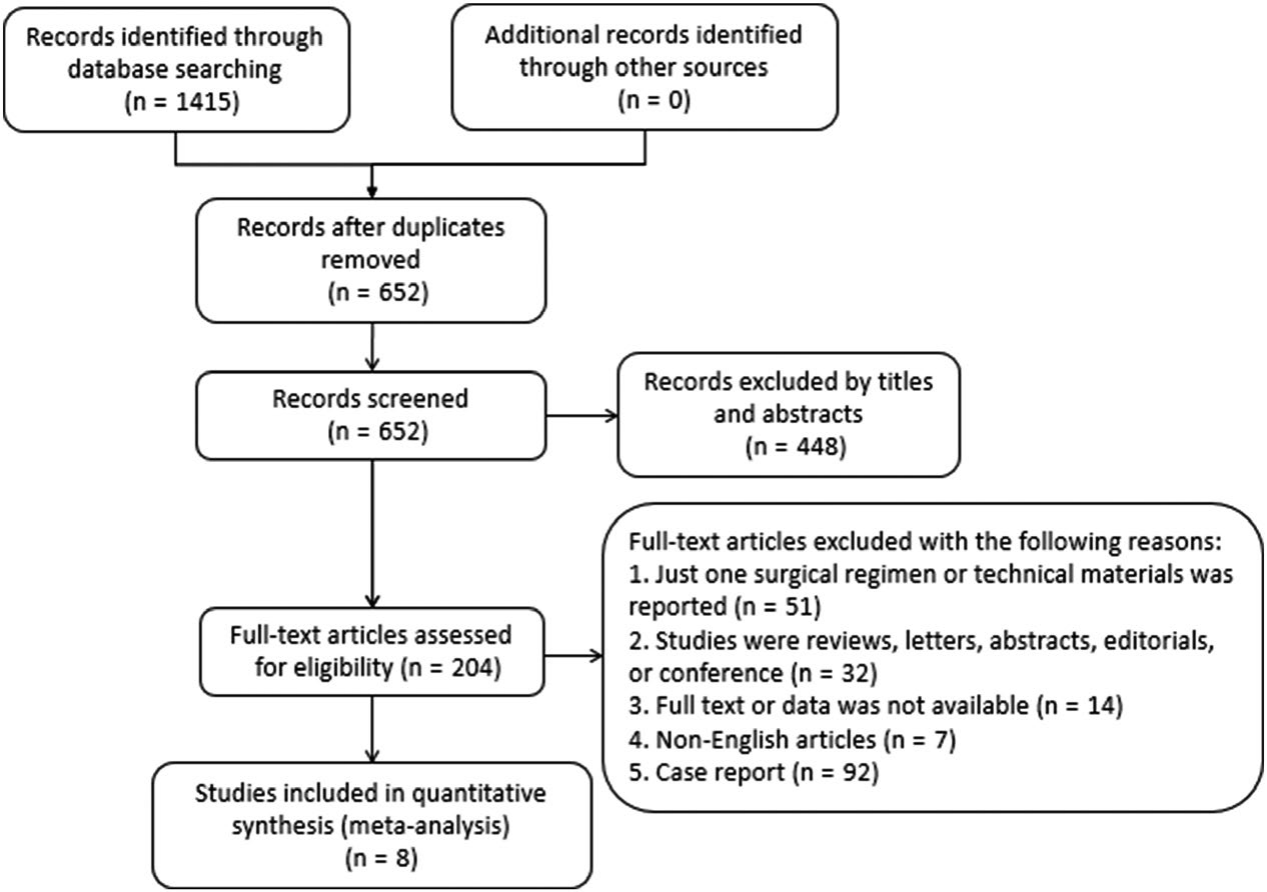
The flowchart of the literature screening, exclusion, and inclusion process

The PRISMA flow diagram shows the details of our literature identification, screening, exclusion, and inclusion processes (Figure 1). The literature search initially yielded a total of 2299 studies. After removing duplicates, 474 studies remained for review of titles and abstracts. A total of 339 potentially eligible studies were included for a full‐text version evaluation after screening titles and abstracts. Among these studies, 331 studies were excluded due to the following reasons: 176 studies were case reports; 77 studies only reported one surgical technique or regimen; 56 studies were editorials, reviews, conference abstracts, or letters; and 22 studies involved other tumors. Following these exclusions, a total of eight studies—including seven cohort studies and one RCT published between 2001 and 2019, collectively representing 1,896 patients—were included in our meta‐analysis.^4^, ^9^, ^10^, ^20^, ^21^, ^22^, ^23^, ^24^

### 3.2 Study characteristics

Table 1 shows the demographic and clinical characteristics of all included studies. All included studies were conducted in Asian countries, most of which were conducted in Japan (seven in Japan, one in Korea). Studies were published between 2001 and 2019. Notably, the most representative study was conducted by multiple central hospitals in Asia and Europe, and this study aimed to investigate the oncological outcomes of patients with LLN ≥7 mm.^4^ Across all included studies, a total of 1896 patients were included, which consisted of 1,461 patients who accepted nCRT+TME vs 435 patients who accepted nCRT+TME+LLND.

**Table 1:** The basic characteristics of studies included in the meta‐analysis

First author, year	Country	Type of study	Years of the study	The treatment of LLN		Age (y)		Gender (male: female)		Height of tumor (cm)		Low clinical tumor stage (I‐II or T1‐2 or Astler‐Coller stage B)		High clinical tumor stage (III‐IV or T3‐4 or Astler‐Coller stage C)		Preoperative nCRT	Indications of nCRT+TME	Indications of nCRT+TME+LLND	Surgical procedure	Pathological LLNM rate	Follow‐up duration (months)		NOS or Jadad score
				nCRT+TME	nCRT+TME+LLND	nCRT+TME	nCRT+TME+LLND	nCRT+TME	nCRT+TME+LLND	nCRT+TME	nCRT+TME+LLND	nCRT+TME	nCRT+TME+LLND	nCRT+TME	nCRT+TME+LLND						nCRT+TME	nCRT+TME+LLND	
Akiyoshi T, 2014	Japan	Retrospective	Between 2004 and 2010	89	38	60 (34‐81)^a^	61 (35‐75)^a^	62:27	28:10	4 (1‐8)^a^	4 (1‐8)^a^	39	0	50	38	45‐50.4 Gy	No LLNM	LLNM (LLN ≥7 mm)	Open or laparoscopy (unilateral or bilateral LLND)	65.80%	47.5 (3.5‐105.4)^a^	47.5 (3.5‐105.4)^a^	7
Ishihara S, 2017	Japan	Retrospective	Between 2003 and 2015	191	31	63.6^b^	60.4^b^	126:65	20:11	4.3^b^	3.4^b^	2	0	189	31	50.4 Gy	No LLNM	LLNM (LLN ≥8 mm)	Open or laparoscopy	51.60%	39.5^a^	39.5^a^	8
Kim HJ, 2017	Korea	Retrospective	Between 2006 and 2013	31	23	—	—	25:6	15:8	—	—	—	—	—	—	45‐50 Gy	LLNM but response to nCRT	LLNM (LLN ≥5 mm)	Unilateral or bilateral LLND	37.70%	34.1 (9‐70)^a^	34.1 (9‐70)^a^	7
Matsuda T, 2018	Japan	Retrospective	Between 2005 and 2016	13	32	68 (40‐79)^a^	64 (39‐76)^a^	9:4	24:8	‐	‐	5	2	8	30	45 Gy	No LLNM	LLNM (LLN ≥8 mm)	Open or laparoscopy (unilateral or bilateral LLND)	23.30%	52^a^	52^a^	7
Nagawa H, 2001	Japan	RCTs	Between 1993 and 1995	22	23	60.1 ± 8.8^b^	59.1 ± 10.1^b^	16:6	17:6	4.8 ± 1.4^b^	4.7 ± 1.6^b^	14^c^	10^c^	8^c^	13^c^	50 Gy	No LLNM	No LLNM	—	—	—	—	1^d^
Ogura A, 2017	Japan	Retrospective	Between 2005 and 2014	220	107	60 (24‐78)^a^	60 (27‐82)^a^	147:73	82:25	4 (0‐10)^a^	4 (1‐10)^a^	0	2	220	105	45‐50.4 Gy/25Gy	No LLNM	LLNM (LLN ≥7 mm)	Laparoscopy (unilateral or bilateral LLND	24.30%	36 (0.5‐124)^a^	36 (0.5‐124)^a^	8
Ogura A, 2019	Japan	Retrospective	Between 2009 and 2013	870	98	—	—	—	—	—	—	—	—	—	—	45‐50.4 Gy/25Gy	LLNM (LLN ≥7 mm)	LLNM (LLN ≥7 mm)	‐	51%	56.5 (55‐58.1)^e^	56.5 (55‐58.1)^e^	7
Watanabe T, 2002	Japan	Retrospective	Between 1985 and 1995	25	53	61.8 ± 9.5^b^	57.9 ± 9.6^b^	16:9	41:12	5.9 ± 1.9^b^	4.6 ± 2.5^b^	—	—	—	—	50 Gy	No LLNM	No LLNM	—	—	—	—	8

Abbreviations: LLN, lateral lymph node; LLND, lateral lymph node dissection; LLNM, clinically suspected lateral lymph node metastasis.; nCRT, neoadjuvant chemoradiotherapy; NOS score, The modified Newcastle‐Ottawa Scale (NOS) score; RCT, randomized controlled trial; TME, total mesorectal excision.

a Data are presented as median (range).

b Data are presented as mean (standard deviation).

c Astler‐Coller stage.

d Data are presented as Jadad score.

e Data are presented as median (interquartile range).

With respect to gender and age, there were no significant differences between the two groups in any individual study. All eight studies adopted the treatment strategy of 5‐fluorouracil‐based long‐course neoadjuvant radiotherapy with a total dose of 45‐50.4 Gy. Two studies also included some patients with short‐course radiotherapy.^4^, ^23^ Usually, the target volume included the lateral region. Notably, six of eight studies performed LLND after nCRT and TME based on the clinically suspected LLNM prior to treatment.^4^, ^9^, ^10^, ^20^, ^21^, ^23^ The criterion of clinically suspected LLNM was mainly based on the LLN short‐axis diameter combined with other imaging features (irregular boundary and heterogeneous signal). On the contrary, patients in six of the eight studies underwent TME only following nCRTm despite no clinically suspected LLNM. In the other two studies, patients in the nCRT+TME group had LLN ≥7 mm or suspected LLN but a response to nCRT.^4^, ^21^ Most studies focused on lower rectal cancer. The distributions of advanced tumor stages (III‐IV, T3‐4, or Astler‐Coller stage C) were similar between the two groups (467 cases in nCRT+TME, 82.5%; 204 cases in nCRT+TME+LLND, 80.3%).^9^, ^10^, ^20^, ^21^, ^22^, ^23^ Both open and laparoscopic unilateral or bilateral LLND were performed. The follow‐up period ranged from 34.1 to 56.5 months.

### 3.3 Quality of included studies

The assessment of methodological quality is shown in Table 1. All seven cohort studies achieved high quality with ≥7 stars.^4^, ^9^, ^10^, ^20^, ^21^, ^23^, ^24^ However, the only RCT study had an unclear risk of bias and relatively low quality (with a score of 1).^22^

### 3.4 Primary outcomes of interest

#### 3.4.1 Local recurrence (LR)

**Figure 2:**
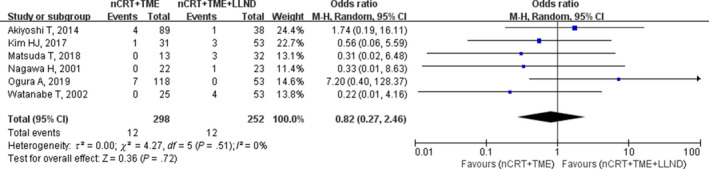
Forest plot of the local recurrence (LR) in the overall population

Six studies reported LR rates for the nCRT+TME group (0%‐5.9%) and nCRT+TME+LLND group (0%‐9.4%), with a pooled mean of 4% (12/298) and 4.8% (12/252), respectively. No significant difference was found between the two groups via meta‐analysis (OR, 0.82; 95% CI, 0.27‐2.46; *P* = .72) without heterogeneity (I^2^ = 0%; Table 2; Figure 2).

**Table 2:** Pooled primary and secondary outcomes of interest of nCRT+TME vs nCRT+TME+LLND in all studies

Outcome	No. of studies	No. of patients		nCRT+TME vs nCRT+TME+LLND			Test of heterogeneity	
		nCRT+TME	nCRT+TME+LLND	OR/HR/MD	95% CI	*P* value	I^2^	*P* value
Primary outcomes								
Local recurrence	6	298	252	0.82	0.27, 2.46	.72	0%	.51
**Local lateral recurrence**	**4**	**429**	**145**	**2.99**	**1.20, 7.74**	**.02**	**0%**	**.82**
Overall survival	3	273	153	0.78	0.32, 1.88	.58	0%	.7
Disease‐free survival	5	387	244	0.94	0.62, 1.43	.77	0%	.91
Secondary outcomes								
Operation time	3	255	162	−138.63	−219.66, −57.60	<.01	81%	<.01
Blood loss	3	255	162	−226.24	−505.76, 53.27	.11	37%	.2
Anastomotic leakage	4	344	200	0.74	0.28, 1.96	.54	0%	.97
Perineal wound infection	4	344	200	0.69	0.39, 1.23	.21	0%	.8
Abdominal wound infection	3	331	168	0.50	0.15, 1.63	.25	22%	.28
Bowel obstruction	4	344	200	1.52	0.52, 4.46	.45	0%	.93
Urinary dysfunction	3	331	168	0.19	0.08, 0.47	<.01	0%	.83

Abbreviations: CI, confidence interval; HR, Hazard Ratio; LLND, lateral lymph node dissection; MD, weighted mean difference; nCRT, neoadjuvant chemoradiotherapy; OR, odds ratio; TME, total mesorectal excision.

With regard to local lateral recurrence, there is significant difference between the nCRT+TME group and nCRT+TME+LLND group without heterogeneity (in bold).

#### 3.4.2 Local lateral recurrence (LLR)

**Figure 3:**
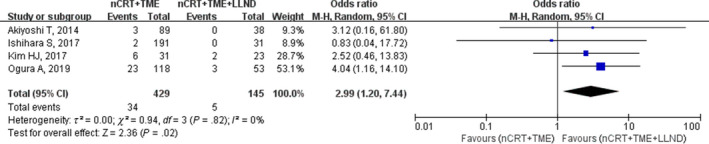
Forest plot of the local lateral recurrence (LLR) in the overall population

Four studies further differentiated LR into LLR and other pelvic‐site recurrences. Among these four studies, the pooled LLR rates were 7.9% (34/429) and 2.9% (5/175) in the nCRT+TME group and nCRT+TME+LLND group respectively. A significant difference was identified between the two groups (OR, 2.99; 95% CI, 1.20‐7.44; *P* = .02) without heterogeneity (I^2^ = 0%; Table 2; Figure 3).

#### 3.4.3 Overall survival (OS)

**Figure 4:**
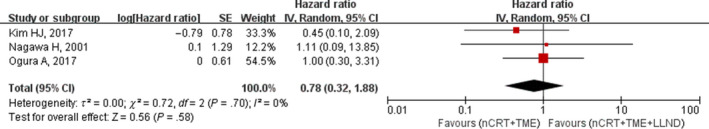
Forest plot of the overall survival (OS) in the overall population

Five studies reported OS outcomes, including 3‐year and 5‐year cumulative OS in the nCRT+TME group and nCRT+TME+LLND group. Meta‐analysis showed that there was no significant difference in OS between the two groups (HR, 0.78; 95%, CI 0.32‐1.88; *P* = .58) with no heterogeneity (I^2^ = 0%; Table 2; Figure 4).

#### 3.4.4 Disease‐free survival (DFS)

**Figure 5:**
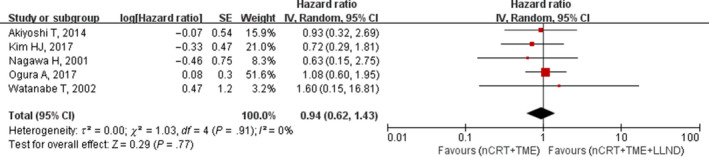
Forest plot of the disease‐free survival (DFS) in the overall population

The DFS outcomes were obtained from five studies also including 3‐year and 5‐year cumulative DFS. Meta‐analysis also showed no significant difference between the nCRT+TME group and nCRT+TME+LLND group with no heterogeneity (HR 0.94, 95% CI 0.62‐1.43, *P* = .77; I^2^ = 0%; Table 2; Figure 5).

#### 3.4.5 The incidence of pathological LLNM

Six studies reported the results of pathological LLNM after nCRT+TME+LLND. The percentage of pathological LLNM ranged from 23.3% to 65.8%, with a pooled mean of 27.8% (121/435; Table 1).

### 3.5 Secondary outcomes of interest

#### 3.5.1 Intraoperative outcomes

**Figure 6:**
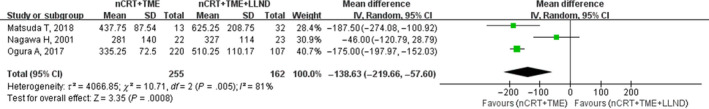
Forest plot of the operation time in the overall population

**Figure 7:**
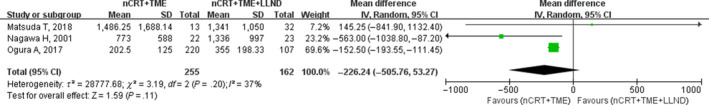
Forest plot of the blood loss in the overall population

Operation time was compared between the nCRT+TME group and the nCRT+TME+LLND group in three studies. Although there was high heterogeneity among these studies, meta‐analysis showed that the use of additional LLND yielded a longer operation time (MD, −138.63; 95% CI, −219.66 to −57.60; *P* < .01; I^2^ = 81%; Table 2; Figure 6). With regard to blood loss, three studies provided data. However, no significant difference was found between the two groups (MD, −226.24; 95% CI, −505.76 to 53.27; *P* = .11). A moderate heterogeneity was identified (I^2^ = 37%; Table 2; Figure 7).

#### 3.5.2 Anastomotic leakage

**Figure 8:**
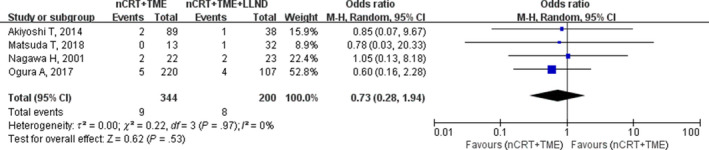
Forest plot of the anastomosis leakage in the overall population

Four studies representing 544 patients reported anastomotic leakage. The anastomotic leakage rate was higher in the nCRT+TME+LLND group (pooled mean, 4%) than in the nCRT+TME group (pooled mean, 2.6%), but meta‐analysis showed no significant difference (OR, 0.73; 95% CI, 0.28‐1.94; *P* = .53; I^2^ = 0%; Table 2; Figure 8).

#### 3.5.3 Abdominal and perineal wound infections

**Figure 9:**
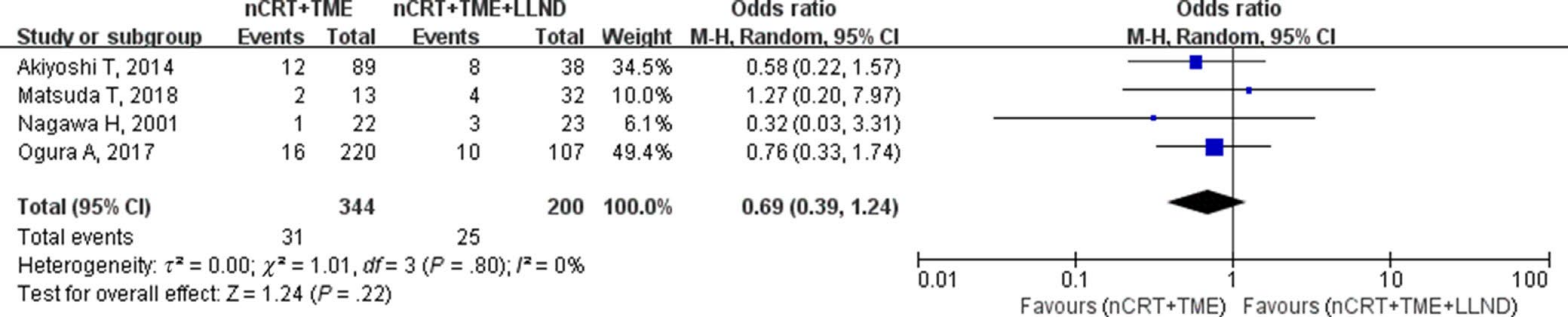
Forest plot of the perineal wound infection in the overall population

**Figure 10:**
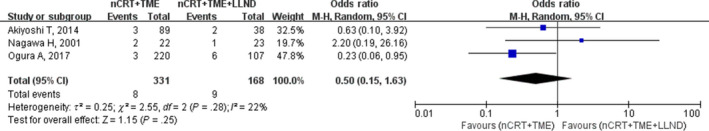
Forest plot of the abdominal wound infection in the overall population

Three studies reported abdominal wound infections, and four studies reported perineal wound infections. The rate of perineal wound infections was similar between the nCRT+TME group and the nCRT+TME+LLND group (9% vs 12.5%; OR, 0.69; 95% CI, 0.39‐1.24; *P* = .22; I^2^ = 0%; Table 2; Figure 9). With respect to abdominal wound infection, no significant difference was found between the two groups (2.4% vs 5.4%; OR ,0.50; 95% CI, 0.15‐1.63; *P* = .25) with low heterogeneity (I^2^ = 22%; Table 2; Figure 10).

#### 3.5.4 Bowel obstruction

**Figure 11:**
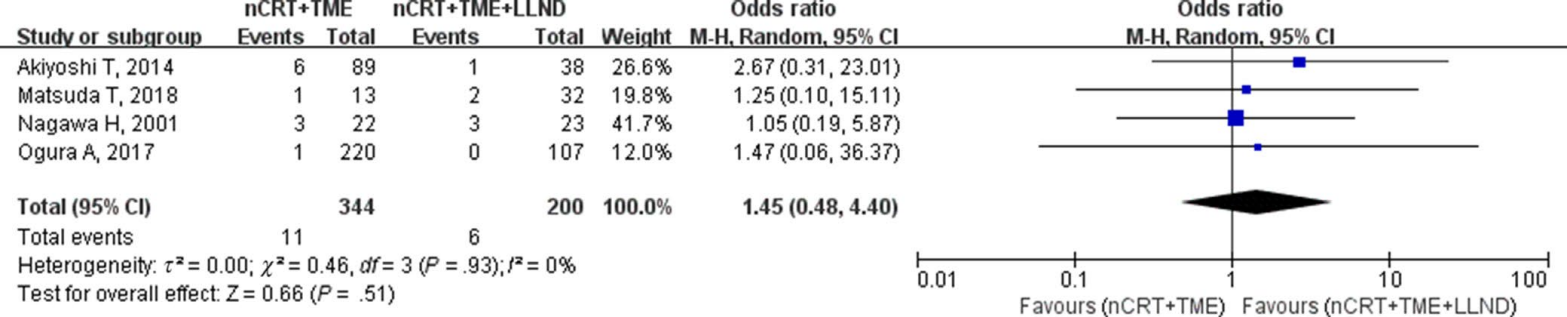
Forest plot of the bowel obstruction in the overall population

The bowel obstruction rate obtained from four studies was 3.2% (11/344) in the nCRT+TME group and 3% (6/200) in the nCRT+TME+LLND group. Meta‐analysis showed no significant difference between the two groups (OR, 1.45; 95% CI, 0.48‐4.40; *P* = .51) without heterogeneity (I^2^ = 0%; Table 2; Figure 11).

#### 3.5.5 Urinary and sexual dysfunction

**Figure 12:**
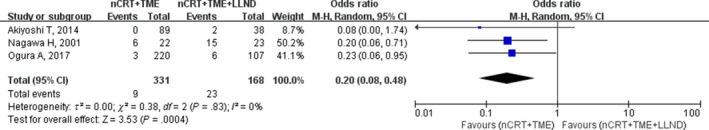
Forest plot of the urinary dysfunction in the overall population

Three studies reported urinary dysfunction, and only one study reported sexual dysfunction. With respect to urinary dysfunction, LLND after nCRT+TME increased the likelihood of this complication compared with that of TME only after nCRT (OR, 0.20; 95% CI, 0.08‐0.48; *P* < .01; I^2^ = 0%; Table 2; Figure 12). The only study involving sexual dysfunction also showed that LLND after nCRT+TME significantly increased the risk of sexual dysfunction.^22^

### 3.6 Heterogeneity analysis

Table 2 shows the results of the heterogeneity tests of the overall analysis. No or low heterogeneity existed in the primary and secondary outcomes of interest, except for operation time and blood loss. Additional LLND after nCRT+TME increased operation time and blood loss. High statistical heterogeneity within these two operative parameters might be caused by many factors, including the retrospective evaluation, non‐uniform definitions of blood loss, and non‐standardized surgery. Due to the limited number of studies, subgroup analysis was not able to be performed to explore the source of the high heterogeneity.

### 3.7 Sensitivity analysis

Sensitivity analysis was performed for pooled outcomes that involved studies published before 2010, with less than 20 patients in each group, or with low quality (Table 3). With regard to operation time, when all studies were considered, high heterogeneity existed (heterogeneity I^2^ = 81%, *P* < .01; Table 2). This was reduced when only high‐quality studies were considered (Heterogeneity χ^2^ = 0.07; *P* = .78). These findings highlighted the importance of good study design and adequate sample size.

**Table 3:** Sensitivity analysis performed for studies comparing nCRT+TME vs nCRT+TME+LLND

Outcome	No. of studies	No. of patients		OR/HR/MD	95% CI	Heterogeneity χ^2^	*P* value
		nCRT+TME	nCRT+TME+LLND				
High‐quality studies							
Local recurrence	5	276	229	0.92	0.29, 2.95	3.91	.42
Overall survival	2	251	130	0.74	0.29, 1.90	0.64	.42
Disease‐free survival	4	365	221	0.97	0.63, 1.51	0.72	.87
Operation time	2	233	139	−175.82	−198.02, −153.62	0.07	.78
Blood loss	2	233	139	−151.99	−193.00, −110.97	0.35	.55
Anastomotic leakage	3	322	177	0.67	0.22, 2.00	0.07	.96
Perineal wound infection	3	322	177	0.73	0.40, 1.34	0.56	.76
Abdominal wound infection	2	309	145	0.34	0.11, 1.03	0.71	.4
Bowel obstruction	3	322	177	1.91	0.46, 7.85	0.23	.89
Urinary dysfunction	2	309	145	0.19	0.05, 0.66	0.38	.54
Studies published after 2010							
Local recurrence	4	251	176	1.2	0.34, 4.28	2.85	.42
Overall survival	2	251	130	0.74	0.29, 1.90	0.64	.42
Disease‐free survival	3	340	168	0.96	0.61, 1.50	0.54	.76
Operation time	2	233	139	−175.82	−198.02, −153.62	0.07	.78
Blood loss	2	233	139	−151.99	−193.00, −110.97	0.35	.55
Anastomotic leakage	3	322	177	0.67	0.22, 2.00	0.07	.96
Perineal wound infection	3	322	177	0.73	0.40, 1.34	0.56	.76
Abdominal wound infection	2	309	145	0.34	0.11, 1.03	0.71	.4
Bowel obstruction	3	322	177	1.91	0.46, 7.85	0.23	.89
Urinary dysfunction	2	309	145	0.19	0.05, 0.66	0.38	.54
Studies with >20 cases per group							
Local recurrence	5	285	220	0.95	0.29, 3.08	3.81	.43
Operation time	2	242	130	−115.61	−241.63, 10.41	10.44	.001
Blood loss	2	242	130	−286.5	−663.77, −90.77	2.84	.09
Anastomotic leakage	3	331	168	0.74	0.27, 2.03	0.22	.9
Perineal wound infection	3	331	168	0.64	0.35, 1.18	0.54	.76
Bowel obstruction	3	331	168	1.58	0.48, 5.26	0.45	.8

Abbreviations: CI, confidence interval; HR, hazard ratio; LLND, lateral lymph node dissection; MD, weighted mean difference; nCRT, neoadjuvant chemoradiotherapy; OR, odds ratio; TME, total mesorectal excision.

### 3.8 Publication bias

**Figure 13:**
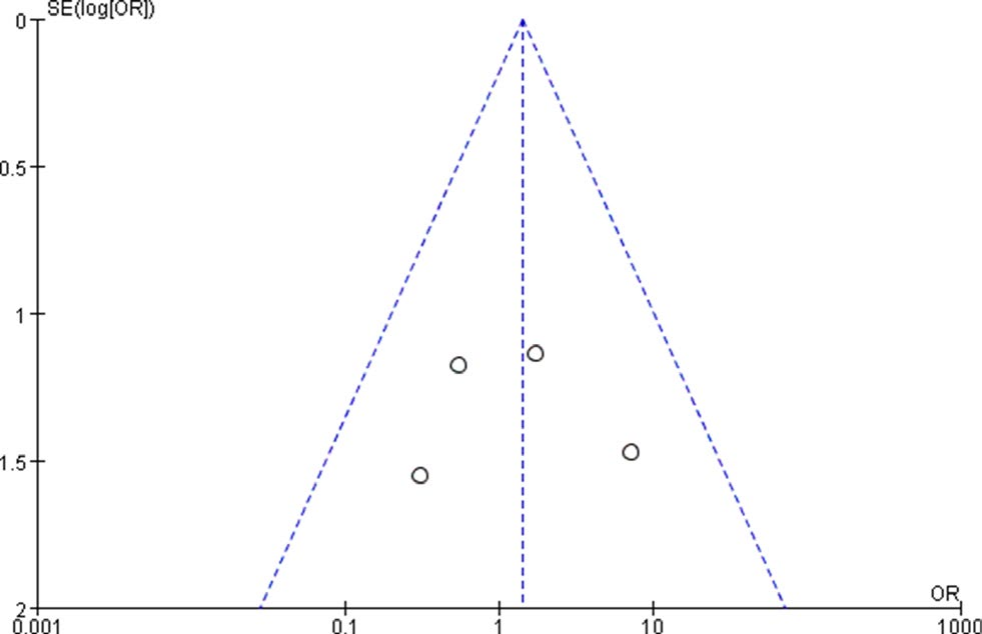
Funnel plot of the local recurrence in the overall population

A funnel plot of the studies used in the meta‐analysis reporting on LR after surgery between the nCRT+TME group and the nCRT+TME+LLND group is shown in Figure 13. All studies resided within the limits of the 95% CI, showing no evidence of obvious publication bias among these studies.

## 4 DISCUSSION

To the best of our knowledge, this is the first meta‐analysis and systematic review to compare nCRT+TME and nCRT+TME+LLND for rectal cancer. According to this study, we found that additional LLND after nCRT significantly reduced the risk of LLR, especially in patients with clinically suspected LLNM, despite inducing longer operation times and increasing the risk of urinary and sexual dysfunction. With respect to OS, DFS, and other postoperative complications, there were no significant differences between the two groups.

Currently, controversy still exists in regard to the treatment of LLN between Western and Eastern practices. The latest Japanese guidelines still recommend prophylactic LLND in patients with low cT3/4 rectal cancer.^3^ In Western countries, several trials have confirmed that increased local control is obtained from preoperative nCRT and TME, resulting in the adoption of nCRT followed by TME as the standard treatment of clinical II/III‐stage rectal cancer.^25^, ^26^, ^27^, ^28^, ^29^ Moreover, compared with prophylactic LLND, this strategy has yielded a similar LLR rate.^30^ Additionally, taking technical difficulties into consideration (especially in obese Western patients and for sexual/urinary dysfunction), it is not surprising that Western surgeons have relied on nCRT to sterilize the lateral compartment.^1^

Recently, increasing evidence has suggested that, in high‐risk patients, nCRT+TME is not sufficient to prevent LLR. Studies with no LLND from Korea showed that the LLR rate increased along with pretreatment lateral‐lymph‐node short‐axis size increasing in patients who underwent nCRT+TME.^5^, ^6^, ^31^ One recent study, which was conducted in a multi‐center in Asian and European countries, demonstrated that LLR is still a significant issue after nCRT+TME, especially in pretreatment LLN ≥7 mm.^4^ Our pooled outcomes also showed that the incidence of LLR in the nCRT+TME group was 7.9%, and this result suggested that nCRT alone cannot completely eradicate LLNM. In contrast, the rate of LLR (3.4%) in the nCRT+TME+LLND group was significantly lower than that in the nCRT+TME group in our present analysis. Our pooled results supported that additional LLND after nCRT+TME could further decrease LLR, especially in patients with clinically suspected LLNM. In addition, according to our meta‐analysis, we found that 27.8% of patients still had pathological LLNM even after preoperative nCRT; these patients had a high‐risk of recurrence if LLND was not performed. Hence, the residual positive LLN after nCRT can be addressed by LLND.

There were no significant differences in the long‐term oncological outcomes (OS and DFS) between the two groups. Some previous studies have reported that long‐term survival outcomes are improved by additional LLND, especially in high‐risk patients with clinically suspected LLNM.^32^, ^33^ However, due to the limited eligible studies that we included, our pooled results did not support that additional LLND could improve survival outcomes. Survival outcomes were affected by many factors, including tumor responses to nCRT, postoperative chemoradiotherapy, and tumor pathological characteristics.^34^ Future treatment strategies for high‐risk patients might be further refined according to the different risks of LLN.^7^

We found that LLND increased operation time and blood loss, especially in patients with preoperative long‐course nCRT. Some studies included in this meta‐analysis even performed bilateral LLND and laparoscopy procedures. However, no studies reported serious intraoperative complications and death within 30 days after operation. Thus, we conclude that LLND after nCRT is relatively safe.

With regard to postoperative complications, there were no significant differences between patients with nCRT+TME and patients with nCRT+TME+LLND, with the exception of urinary dysfunction. This meta‐analysis showed that LLND significantly increased the incidence of urinary dysfunction, which is similar to the results of another early meta‐analysis comparing TME alone vs TME+LLND.^1^ The pooled result was mainly affected by the only RCT study with low quality, and no more details of operation could be identified in the primary study.^22^ We hypothesize that—with the development of standardized surgical procedures, autonomic nerve preservation techniques, and minimally invasive surgery—postoperative genitourinary function will be largely improved.^11^, ^12^, ^35^, ^36^, ^37^

Our meta‐analysis had some limitations. First, the available studies were relatively small, and the majority of the included studies were retrospective. However, no high heterogeneities existed in the pooled outcomes of interest, except for operation time. Furthermore, according to our sensitivity analysis, we identified the source of heterogeneity on operation time. Second, two eligible studies included some patients with short‐course radiotherapy, which might have introduced a bias. However, we were unable to perform further subgroup analysis due to insufficient data. Third, most of the included studies derived from Japan. With regard to LLR, four studies reported its incidence, and two studies included patients from the same institution.^4^, ^9^ However, patients in these two studies derived from different periods (2004‐2010 and 2009‐2013). Therefore, we argue that these partially overlapped data did not affect the validity of our conclusion. Finally, further research from different regions of the world is needed to confirm our present findings.

In conclusion, our study showed that the incidence of pathological LLNM in patients with clinically suspected LLNM after CRT+TME was still relatively high. For these patients, LLND after nCRT+TME contributed to decreasing LLR, while it prolonged operation time and increased the risk of urinary dysfunction (which might be improved by minimally invasive procedures). We believe that our results provide another treatment option for such high‐risk patients.

## CONFLICT OF INTEREST

We declare no conflicts of interest.

## AUTHOR CONTRIBUTIONS

Xuyang Yang, Shuo Yang, Tao Hu, and Chaoyang Gu contributed equally to this work. Ziqiang Wang and Zongguang Zhou conceive this meta‐analysis. Xuyang Yang, Shuo Yang, Tao Hu, and Chaoyang Gu perform the research, collect and analyze the data, draft the article. Mingtian Wei, and Xiangbing Deng analyze the data and revise critical intellectual content. All authors read and approve the final manuscript and agree to be accountable for all aspects of work to ensure that questions regarding accuracy and integrity investigated and resolved.

## Data Availability

The data that support the findings of this study are available from the corresponding author upon reasonable request.
